# Supplementary material for: Ultralong 100 ns spin relaxation time in graphite at room temperature
Source: Nat Commun. 2023 May 17;14:2831. doi: 10.1038/s41467-023-38288-w (PMC10192359; doi:10.1038/s41467-023-38288-w)
Supplement: Supplementary file 1 — Supplementary Information [file 41467_2023_38288_MOESM1_ESM.pdf]

SUPPORTING INFORMATION

This supporting information provides additional band structure calculations and ESR data, the general treatment of Yafet's theory for uniaxially anisotropic materials; in particular individual ESR spectra, the X-band ESR signal intensity as a function of temperature, the ESR line anisotropy, the angular dependence of the g-factor and linewidth, and temperature dependence of the g-factor.

Supporting Information A: Band structure in the whole BZ

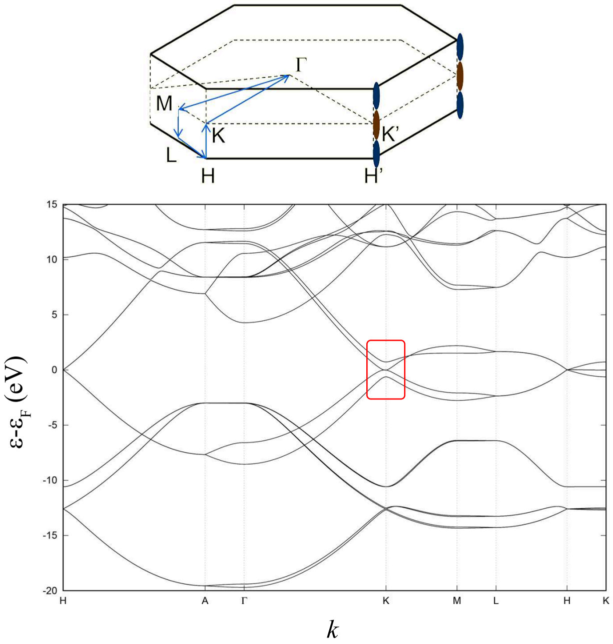

FIG. S1. The top figure is the Brillouin zone of graphite with high symmetry points noted. Colored parts show the Fermi surface with two major charge carrier types: **electrons** (brown) and **holes** (blue). The bottom image displays the band structure. The red rectangle denotes the vicinity of the **K** point, which is relevant to understanding the relaxation properties of the material.

The top part of Fig. S1. displays the Brillouin zone (BZ) of graphite with high symmetry points noted. The shape of the Fermi surface resembles a "parlour candy" (popular in central Europe) with two types of charge carriers, electrons near $\mathbf{K}(\mathbf{K}')$ and holes near $\mathbf{H}(\mathbf{H}')$. The bottom part shows the band structure for the entire BZ in the directions noted above. The relevant part to understand spin physics is highlighted with a red rectangle. Our results return previous reports[1–3] when the fine details are discarded.

Supporting Information B: Additional ESR data

Fig. S2. shows the temperature-dependent X-band ESR intensity data as well as some individual spectra. The ESR intensity can be well fitted with a sum of a Curie ($\chi \propto 1/T$) and a Pauli-like ($\chi \propto$ constant) spin-susceptibilities. The earlier corresponds to some localized paramagnetic impurities in the graphite (e.g., isolated paramagnetic Fe ions) and the latter to the signal of the itinerant electrons in graphite. Fe ions are known to be a common impurity in graphite which influences the magnetic properties[4,5]. We note that in principle, the contribution of the itinerant electrons is expected to drop by about a factor of two between 300 K and 20 K due to the decrease in the charge carrier density of states[1,6], however, the simultaneous increase of the spin-susceptibility due to the impurities and some instrumental-related uncertainties (e.g., the change in the resonator quality factor with temperature) prevents the observation of this effect.

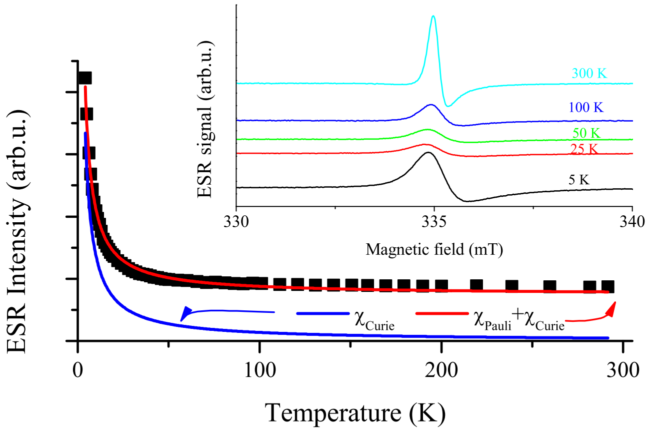

FIG. S2. ESR intensity for the as-received sample for $B_{\|}$ in the $5-300$ K temperature range, inset shows the ESR spectra for a few temperatures. Solid curves show the fitted signal intensity with a Pauli (constant) and Curie ($\sim 1/T$) contributions. Inset shows individual ESR spectra.

The ESR signal is constant in the $50-300$ K temperature range, which is followed by a Curie-like increase towards lower temperatures thus a constant plus Curie signal (i.e., with a zero Weiss temperature) fits well to the experimental data as shown in the figure. The annealing does not affect significantly the low temperature Curie upturn. The relation between the ESR signal intensity and the measured spin-susceptibilities is affected by the finite penetration of the microwaves into the sample. The resistance in the a, b plane decreases by a factor 4 between 300 K and 50 K and decreases roughly linearly below this temperature[6]. This means that the microwave penetration depth changes by only a factor of two between 300 and 50 K. This leads us to conclude that at least in this temperature range, the ESR signal is proportional to the spin-susceptibility of the sample, but it underestimates that below 50 K.

A similar ESR signal increase is often encountered when

studying carbon based metals (the examples include alkali fullerides[7], carbon nanotubes[8], and boron-doped diamond[9]), and this effect is due to a common resonance of the itinerant electrons and a minute amount (few ppm) of localized paramagnetic moments. The latter can arise from either transition metal contaminants in graphite or from dangling bonds on the graphene edges.

Graphite is particularly prone to this effect as it has a very low conduction electron spin-susceptibility ($\chi_{\text{Pauli}} = 3.5 \cdot 10^{-8}$ emu/cm^3, Ref.10) due to the low density of states on the Fermi surface (0.01 states/eV $\cdot$ atom, Ref.11), which corresponds to the Curie spin-susceptibility of about 5 ppm $S = 1/2$ spins at 10 K. It is characteristic for a common resonance signal[12] that the observed quantities, linewidth and g-factor, are averages, weighted with the corresponding spin-susceptibilities, of the respective parameters[12]. Clearly, at low temperatures, the spin-susceptibility of the paramagnetic centers prevails over that of the itinerant electrons in the X-band ESR data.

This dominance of the paramagnetic impurities in the ESR signal at low temperatures matches well the observed annealing dependence of the ESR linewidth: the high-temperature annealing can affect the concentration of these centers and their coupling to the localized spins either by neutralizing them or by inducing a diffusion. Much as the low-temperature behavior appears to be dominated by defects, the important conclusion from our measurements is that the behavior of the ESR linewidth above $\sim 50 - 100$ K is intrinsic to graphite and is unaffected by impurities.

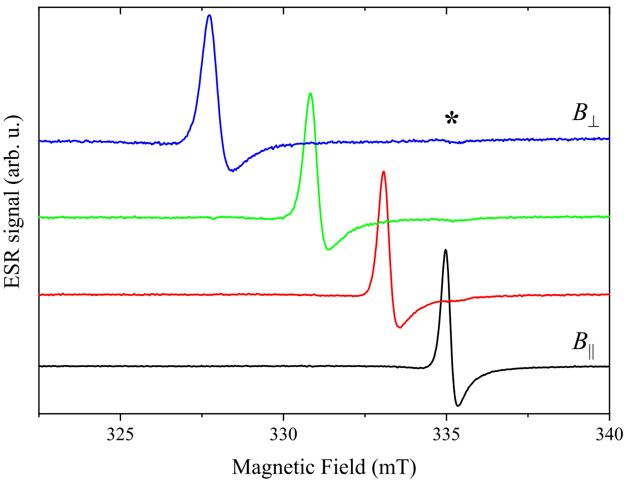

FIG. S3. Angular dependence of the X-band ESR line at room temperature for the vacuum annealed sample. Note the shift of the resonance line to lower resonance fields when $B_\perp$, which indicates a higher g-factor. Also, note the line-broadening for this direction. Asterisk indicates a small impurity line around $g = 2$, which is effectively decoupled from the ESR signal of itinerant electrons for the $B_\perp$ direction.

In Fig. S3., we show the angular dependent X-band ESR data for a HOPG sample that was thermally annealed in vacuum, prior to the measurement. When $B_\perp$, the line shifts toward lower resonance magnetic fields and broadens. Asterisk in the figure indicates a small impurity line around $g = 2$, which becomes visible for this orientation as the resonance field difference effectively decouples it from the ESR signal of the itinerant electrons.

In Fig. S4., we present the angular dependence of the g-factor and linewidth for the vacuum annealed sample (the properties are identical for the as-received sample). A simple sinusoidal fit is shown for both types of data, which appropriately explains the data. It also confirms that the studied HOPG samples contain single crystallites, which are well aligned with respect to the crystalline c axis.

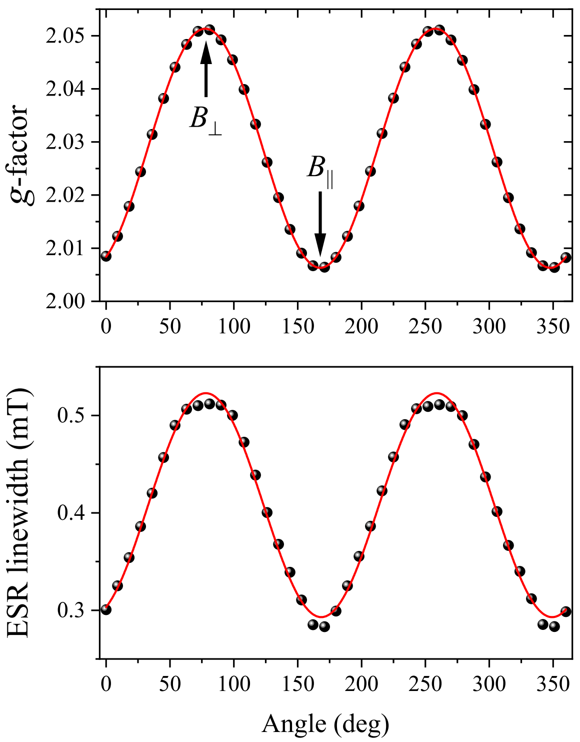

FIG. S4. Angular dependence of the HOPG g-factor and linewidth. The angle scale is arbitrary and arrows indicate the directions of the magnetic field along the major crystalline axes. A sinusoidal fit is shown for both kinds of data.

The temperature dependent g-factor data, presented in Fig. S5, confirms the earlier observed anomalous behavior[13–16]: for $B_\parallel$, it is temperature independent and $g_\parallel \approx 2.005(2)$, however when $B_\perp$, the g-factor is strongly temperature dependent and has a larger value, $g_\perp(300\text{ K}) = 2.05$ and $g_\perp(50\text{ K}) = 2.14$. As graphite is known to possess a sizeable macroscopic diamagnetism, which is strongly angular and temperature dependent, we discuss its influence on the g-factor. We first note that when a material has macroscopic diamagnetism, a larger external field is required to fulfill the resonance conditions, i.e., the g-factor shifts to *negative* values. A recent study on the graphite macroscopic susceptibility[16]

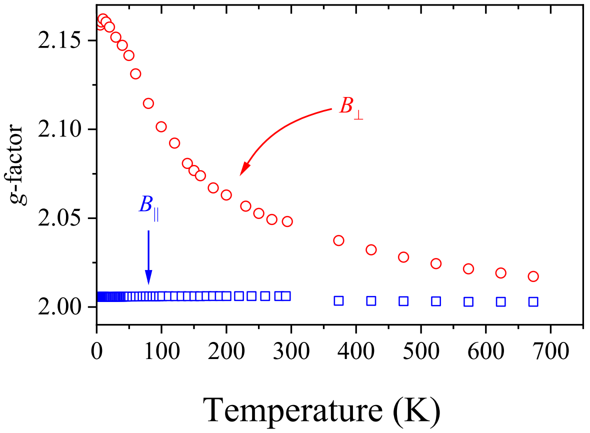

FIG. S5. Extreme anisotropy and temperature dependence of the g-factor for the two orientations.

found $\chi_\perp = -4.2 \cdot 10^{-5}$ emu/cm^3 at 300 K and $\chi_\perp = -6.4 \cdot 10^{-5}$ emu/cm^3 at 50 K. For $B_\parallel$, the macroscopic susceptibility is about 50 times smaller. In general, the g-factor shift due to macroscopic magnetism is $\Delta g/g_0 = (g - g_0)/g_0 = 4\pi\chi$ (the free-electron g-factor is $g_0 = 2.0023$), where χ is the volume susceptibility in emu/cm^3 units, which gives for graphite a maximum g-factor shift due to macroscopic magnetism of $-8 \cdot 10^{-4}$. This value has the opposite sign and is about 2 orders of magnitude smaller than the experimentally observed $\Delta g_\perp$. This leads us to conclude that the macroscopic susceptibility of graphite plays no significant role in the observed g-factor and given the temperature dependence of χ, we chose to neglect it in the analysis below.

In Fig. S6., we show the temperature dependence of HOPG for $B_\perp$ for the as-received and thermally annealed sample. As for this direction, the main ESR line of itinerant electrons is decoupled from impurities (due to different g-factors[12]), the impurity signal is clearly observed as a function of temperature around the $g = 2$ resonance field. The ESR signal of itinerant electrons shifts to lower resonance fields due to the temperature-dependent g-factor and broadens. The excessive broadening is probably the reason why the signal is not observable below 25 K. According to the data, only the impurity signal lineshape is affected by the thermal annealing and the properties of the ESR signal of itinerant electrons is unaffected.

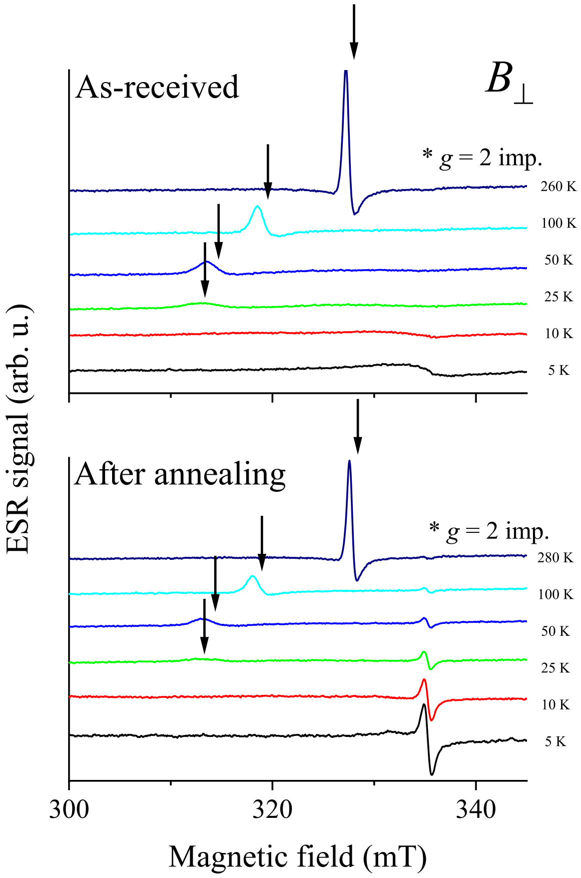

FIG. S6. Temperature dependence of the ESR spectra for $B_\perp$ for the as-received sample and after thermal annealing. Note that the thermal annealing only affects the paramagnetic impurity signal around $g = 2$ (marked by an asterisk). Note the strongly shifting ESR signal of itinerant electrons, which broadens at low temperatures and is not visible below 25 K.

Supporting Information C: ESR spin-relaxation rate for arbitrary magnetic field orientation

A geometrical argument leads to the following relaxation rates when the magnetic field is rotated from the c-axis (θ is the polar angle measured from c):

$$(T_1)^{-1}(\theta) \propto \left(1+\cos^2\theta\right) \cdot \delta B_\parallel^2 + \sin^2\theta \cdot \delta B_\perp^2$$

$$(T_2)^{-1}(\theta) \propto \frac{\left(1+\sin^2\theta\right) \cdot \delta B_\parallel^2 + \cos^2\theta \cdot \delta B_\perp^2}{2} + \frac{\delta B_\parallel^2 + \delta B_\perp^2}{2}.$$

Supporting Information D: Saturated ESR experiments

In principle, T_1 and T_2 could be determined from spin-echo experiments when both quantities are larger than a few 100 ns, which is not the case herein. The possibility to determine T_1 from saturated ESR experiments is a direct consequence of the solution to the Bloch equations[17] when the irradiating AC magnetic field strength, B_1 is large such that the so-called saturation factor $s = \gamma^2 B_1^2 T_1 T_2$, is large. The solu-

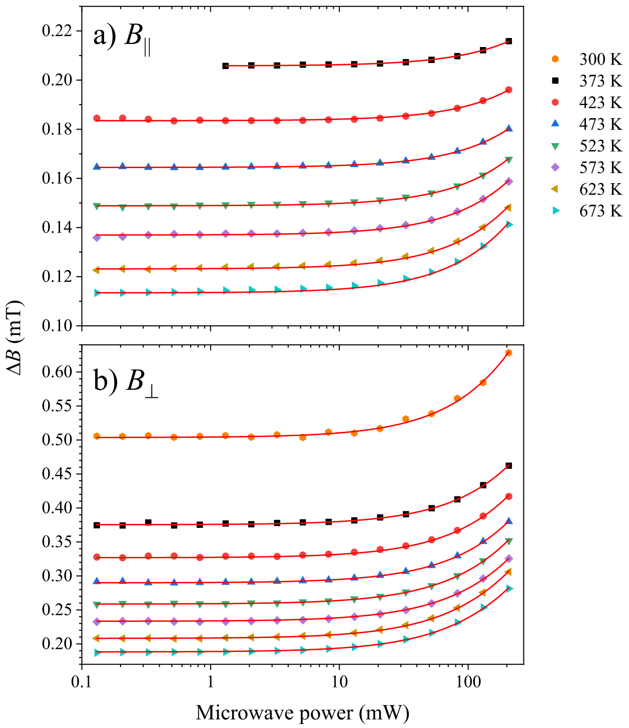

FIG. S7. Microwave power dependence of the ESR linewidth for both geometries as a function of temperature. Solid curves show fits as explained in the text. Note the larger effect of saturation for the $B_\perp$ orientation, which indicates a long T_1.

tions indicate[18] that both the ESR signal intensity, I, and the ESR linewidth, ΔB are affected under saturation as

$$I \propto \frac{I_0 \cdot B_1}{\sqrt{1+s}}, \tag{D1}$$

$$\Delta B = \Delta B_0 \cdot \sqrt{1+s}. \tag{D2}$$

Due to technical reasons, measurement of the saturated linewidth provides a more accurate estimate of T_1 than measurement of the ESR signal intensity.

In. Fig. S7., we show the ESR linewidth under saturation, i.e., as a function of microwave irradiation power for various temperatures for both orientations of the magnetic field. We note that care was taken to avoid sample heating by microwave-induced eddy currents with the use of a large flux of thermalized N_2 exchange gas. Besides, were sample heating present, it would *decrease* the observed linewidth due to temperature dependence of the linewidth. Therefore the observed upturn in the linewidth for large irradiating microwave powers is a clear sign of a sizeable saturation effect. This effect is more pronounced for the $B_\perp$ orientation, which hints that the corresponding T_1 is longer than for $B_\parallel$.

The irradiating microwave power, p, can be converted to B_1 according to the $B_1 = 0.2\ \mathrm{mT}\sqrt{\frac{pQ_\mathrm{L}}{Q_0}}$ relation which is given by the producer of the microwave cavity while considering the measured loaded quality factor, Q_L, of the cavity with respect to the unloaded value of Q_0. Then, Eq. (D2) is used to fit the data in Fig. S7., with T_1 being essentially the only free parameter as the value of T_2 is fixed by the non-saturated value of the linewidth, ΔB_0.

[1] McClure, J. W. Band Structure of Graphite and de Haas-van Alphen Effect. *Phys. Rev.* **108**, 612–618 (1957).
[2] Ooi, N., Rairkar, A. & Adams, J. B. Density functional study of graphite bulk and surface properties. *Carbon* **44**, 231 – 242 (2006).
[3] Grüneis, A. *et al.* Tight-binding description of the quasiparticle dispersion of graphite and few-layer graphene. *Phys. Rev. B* **78**, 205425 (2008).
[4] Sepioni, M., Nair, R. R., Tsai, I.-L., Geim, A. K. & Grigorieva., I. V. Revealing common artifacts due to ferromagnetic inclusions in highly oriented pyrolytic graphite. *Europhysics Letters* **97**, 47001 (2012).
[5] Mittal, R. *et al.* Neutron-Irradiation Induced Magnetization and Persistent Defects at High Temperatures in Graphite (2020). 2008.04032.
[6] Soule, D. E. & McClure, J. W. Band structure and transport properties of single-crystal graphite . *Journal of Physics and Chemistry of Solids* **8**, 29 – 35 (1959).
[7] Nemes, N. M. *et al.* Conduction-electron spin resonance in the superconductor K_3C_{60}. *Phys. Rev. B* **61**, 7118–7121 (2000).
[8] Szirmai, P. *et al.* Doped carbon nanotubes as a model system of biased graphene. *Phys. Rev. B* **96**, 075133 (2017).
[9] Szirmai, P. *et al.* Observation of conduction electron spin resonance in boron-doped diamond. *Phys. Rev. B* **87**, 195132 (2013).
[10] Dresselhaus, M. S. & Dresselhaus, G. Intercalation compounds of graphite. *Adv. Phys.* **51**, 1–186 (2002).
[11] Galambos, M. *et al.* Identifying the electron spin resonance of conduction electrons in alkali doped SWCNTs. *Phys. Stat. Sol. B* **246**, 2760–2763 (2009).
[12] Barnes, S. E. Theory of electron spin resonance of magnetic ions in metals. *Adv. Phys.* **30**, 801–938 (1981).
[13] Wagoner, G. Spin Resonance of Charge Carriers in Graphite. *Phys. Rev.* **118**, 647–653 (1960).
[14] Singer, L. S. & Wagoner, G. Electron Spin Resonance in Polycrystalline Graphite. *The Journal of Chemical Physics* **37**, 1812–1817 (1962).
[15] Matsubara, K., Tsuzuku, T. & Sugihara, K. Electron spin resonance in graphite. *Phys. Rev. B* **44**, 11845–11851 (1991).
[16] Huber, D. L., Urbano, R. R., Sercheli, M. S. & Rettori, C. Fluctuating field model for conduction electron spin resonance in graphite. *Phys. Rev. B* **70**, 125417 (2004).
[17] Slichter, C. P. *Principles of Magnetic Resonance* (Spinger-Verlag, New York, 1989), 3rd ed. 1996 edn.
[18] Portis, A. M. Electronic Structure of F Centers: Saturation of the Electron Spin Resonance. *Phys. Rev.* **91**, 1071 (1953).
